# Metallic Strontium as a Precursor of the Al_2_O_3_/SrCO_3_ Xerogels Obtained by the One-Pot Sol–Gel Method

**DOI:** 10.3390/gels8080473

**Published:** 2022-07-27

**Authors:** Eliza Romanczuk-Ruszuk, Bogna Sztorch, Zbigniew Oksiuta, Robert E. Przekop

**Affiliations:** 1 Institute of Biomedical Engineering, Faculty of Mechanical Engineering, Bialystok University of Technology, Wiejska 45C Street, 15-351 Bialystok, Poland; e.romanczuk@pb.edu.pl (E.R.-R.); z.oksiuta@pb.edu.pl (Z.O.); 2 Centre for Advanced Technologies, Adam Mickiewicz University in Poznan, Uniwersytetu Poznanskiego 10 Street, 61-614 Poznan, Poland; bogna.sztorch@amu.edu.pl

**Keywords:** sol–gel, metallic precursor, SrCO_3_, xerogels, alumina, binary gels, one-pot

## Abstract

Two series of binary xerogel systems of Sr/Al with molar ratios of 0.1, 0.25, 0.5, and 1.0 were synthesized by the sol–gel technique with metallic strontium component as a precursor. The influence of the metallic precursor on the properties of the final xerogel was determined. The properties of the gels were determined on the basis of X-ray powder diffraction (XRD), thermogravimetric analysis (TGA), low temperature nitrogen adsorption, transmission, and scanning electron microscopy with Energy Dispersive X-ray Spectroscopy (TEM, SEM, and SEM/EDS). The Al_2_O_3_/SrCO_3_ xerogels were tested as supports for platinum catalysts. Hydrogen chemisorption was used to determine the platinum dispersion of the Pt/Al_2_O_3_-SrCO_3_ systems. The original method of synthesis allows to obtain highly dispersed and stable strontium carbonate phases that allow for obtaining a high (42–50%) dispersion of platinum nanoparticles.

## 1. Introduction

Xerogels are described as porous systems obtained by drying wet gels and retaining their porous structure after drying [1]. The advantages of xerogels are thermal stability, large surface area, and porosity. Xerogels are biocompatible and nontoxic and can be easily modified [2]. Silica xerogels are the most popular xerogels and can be used as fillers for polymer composites. They are characterized by low density, high thermal stability, low thermal conductivity, and good hydrophobic properties [3].

It is well-known that metal oxides characterized by large specific surfaces and thermal stability, such as Al_2_O_3_, SiO_2_, TiO_2_, and CeO_2_, can be used as catalyst supports. In addition to the well-recognized oxides, oxides of the alkaline earth metal groups are also used for catalytic processes. Strontium oxide (SrO) is an example of an oxide of the alkaline earth metal group. It can catalyze numerous synthetic reactions, like nitroaldol reactions, selective oxidation of propane, and oxidative coupling of methane [4,5,6]. Strontium has a lower electronegativity among metals from Group II of the Periodic Table. Therefore, strontium oxide has a higher basic strength compared to other group II oxides. The electronegativity increases in the order MgO < CaO < SrO < BaO [4]. Nevertheless, there is limited research on using SrO as a catalyst. The problem is related to the preparation and use of SrO as the base catalyst, as there are difficulties in the preparation of SrO [7].

Strontium carbonate (SrCO_3_) takes advantage of the production of X-ray tubes, hard magnets, ceramics, and special glasses [8]. Before or during the high-temperature production processes, strontium carbonate (SrCO_3_) is decomposed into strontium oxide (SrO) and carbon dioxide (CO_2_). Strontium carbonate decomposes into strontium oxide and carbon dioxide during calcination at a temperature above 1000 °C (1273 K) in atmospheric conditions [9,10]. Recently, much work has focused on the use of carbonate as a catalyst support. It is worth noting that carbonate is not a typical catalyst support. In the work of Omat et al. [11] it was found that cobalt deposited on SrCO_3_ showed exceptional activity for the dry reforming of methane. Another study investigated the selectivity and reactivity of cobalt deposited on an alkaline earth metal carbonate for the catalytic preferential oxidation of CO [12,13]. The catalytic properties of Co/SrCO_3_ were greater compared to cobalt catalysts supported on popular metal oxides. Moreover, Co/SrCO_3_ showed the best productivity. In Iida et al. [14], the reforming of the catalytic activity of toluene of Ru/SrCO_3_-Al_2_O_3_ and Ru/Al_2_O_3_ catalysts in steam was compared. The research shows that the carbonate catalyst shows higher activity.

The aim of this study was to determine the properties of Al_2_O_3_/SrCO_3_ xerogels obtained from metallic strontium. Therefore, a new synthesis approach using an Al_2_O_3_/SrCO_3_ using the sol–gel method is presented. In the proposed method, the precursors of one of the matrix components were used in metallic form. Our previous work has indicated that the introduction of a metallic precursor changes the properties of the final product [15].

The advantage of using the sol–gel synthesis is the purity of the materials obtained without inorganic admixtures and residual ion content [16].

## 2. Results and Discussion

### 2.1. X-ray Powder Diffraction

**Figure 1:**
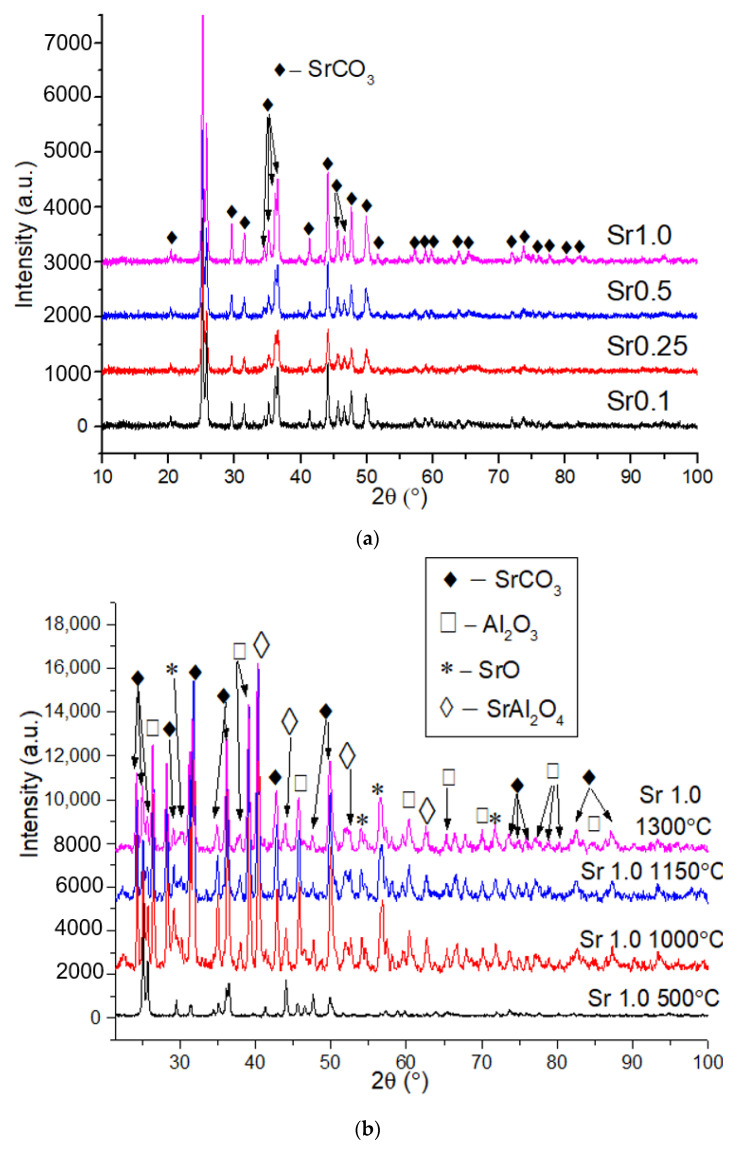
XRD patterns of Al_2_O_3_/SrCO_3_ systems: (**a**) annealed at 500 °C (773 K) with Sr molar ratios of 0.1, 0.25, 0.5 and 1.0; (**b**) Sr 1.0 molar ratio at different temperature.

Figure 1 presents X-ray diffraction patterns of Al_2_O_3_/SrCO_3_ systems annealed in 500 °C (773 K) with different strontium (Sr) content and Sr1.0 annealed in different temperatures. The XRD results of Al_2_O_3_/SrCO_3_ samples with a different amount of strontium indicate only the presence of SrCO_3_ in the structure. The appearance of SrCO_3_ in the XRD results can be explained by the strontium acetate decomposition, which takes place at 400–480 °C (673–753 K) [17]. As the Sr content increases, there is a change in the broadening and intensity of the peaks (Figure 1a). As expected, the highest intensity of the peaks in the sample with an Al/Sr molar ratio of 1.0 was detected. The XRD plot of the Sr1.0 annealed at different temperatures varies with the temperature operated (Figure 1b). XRD analysis of Sr1.0 materials annealed at 1000 °C (1273 K), 1150 °C (1423 K), and 1300 °C (1573 K) shows the same phases, but the peaks differ in intensity. The difference in the intensity of the peaks after different annealing times may be due to the amount of SrCO_3_ crystallized [18]. In each of these materials one or more of the following compounds was identified: SrCO_3_, Al_2_O_3_, SrO, or SrAl_2_O_4_. The appearance of SrAl_2_O_4_ can be explained by the interfacial reaction between SrCO_3_ and Al_2_O_3_ at a temperature above 500 °C (773 K) and the diffusion of Al^3+^ ions in the SrCO_3_ lattice, which causes the formation of SrAl_2_O_4_ [19]. Moreover, the presence of SrAl_2_O_4_ may be related to the decomposition of strontium carbonate to SrO at higher temperatures and to the reaction with Al_2_O_3_, which is represented as follows [20]:

SrCO_3_ → SrO + CO_2_ (1)

SrO + Al_2_O_3_ → SrAl_2_O_4_ (2)

The presence of SrO in samples at temperatures above 1000 °C (1273 K) can be explained by the fact that in the temperature range 900–1175 °C (1173–1448 K) the equilibrium state moves toward the carbonate decomposition [21]. The SrO-CO_2_-SrCO_3_ equilibrium diagram by Rhodes et al. [22] shows that at temperatures below 900–1000 °C (1173–1272 K) the equilibrium moves toward the formation of SrCO_3_; furthermore, at higher temperatures the possibility of SrCO_3_ decomposition increases [21,22,23].

### 2.2. SEM, TEM, and EDS Analysis

**Figure 2:**
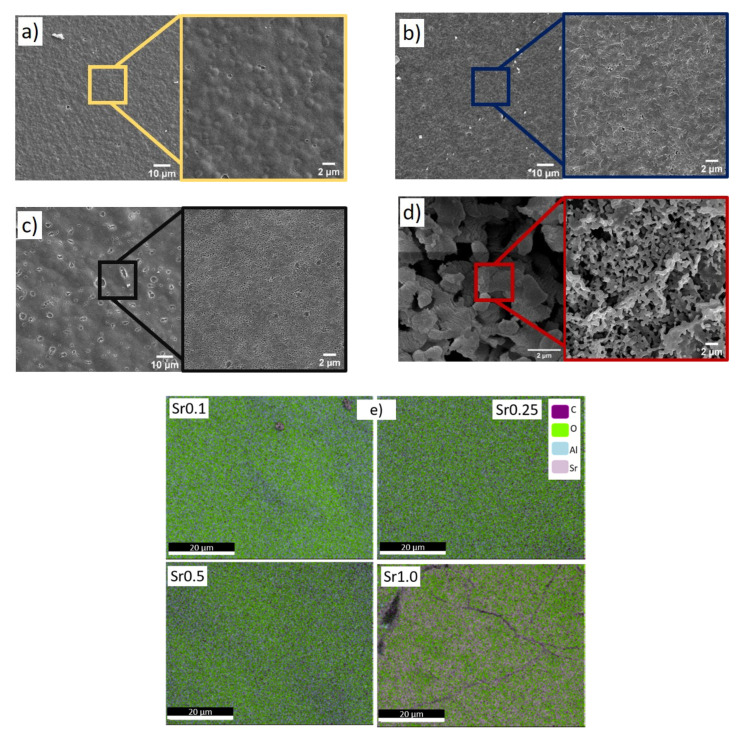
SEM micrographs of the Al_2_O_3_/SrCO_3_ xerogels with various molar ratios: (**a**) Sr0.1, (**b**) Sr0.25, (**c**) Sr0.5, (**d**) Sr1.0, and (**e**) SEM-EDS analysis.

Figure 2 shows the surface of the samples with different molar ratios of Sr before annealing. Note that the surfaces of the tested materials vary depending on the amount of Sr. The SEM image of the Sr0.1 sample surface presents a granular structure. The Sr1.0 sample has a completely different structure compared to the other tested materials. The SEM EDS analysis (Figure 2e) shows that the elements in the examined xerogels systems decompose regularly. Oxygen clusters and a higher strontium content are observed in the Sr1.0 sample compared to the other tested xerogels.

**Figure 3:**
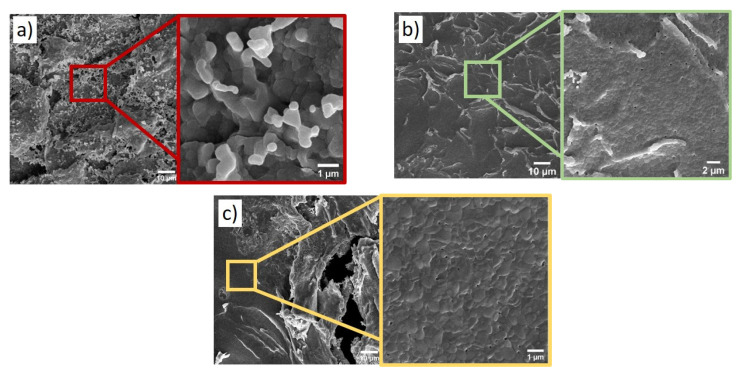
SEM micrographs of surface Al_2_O_3_/SrCO_3_ xerogels with the 1.0 Sr molar ratio annealed at: (**a**) 1000 °C (1273K), (**b**) 1150 °C (1423K), and (**c**) 1300 °C (1573K).

Figure 3 presents a comparison of the SEM micrographs of the Al_2_O_3_/SrCO_3_ samples with Sr to an Al ratio of 1.0 annealed at different temperatures. The structure of the gel after annealing at 1000 °C (1273 K) (Figure 3a) is non-homogeneous. The surface of the gels after annealing at 1150 °C (1423 K) and 1300 °C (1573 K) is similar (Figure 3b,c, respectively). In the material after annealing at 1150 °C (1423 K) and 1300 °C (1573 K), a monolithic structure with a uniform composition distribution without any traces of crystallization of the strontium phases is observed. In the xerogel annealed at 1000 °C (1273 K), a hierarchical structure with the effect of phase aggregation can be observed.

**Figure 4:**
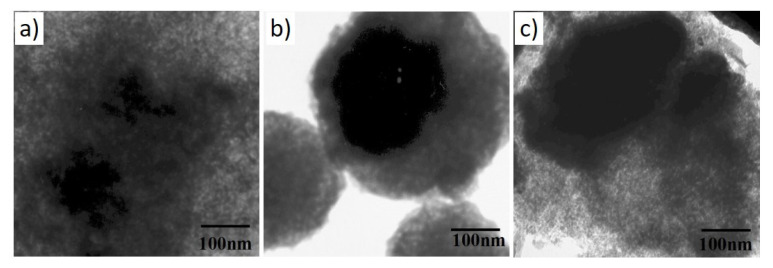
TEM images of Al_2_O_3_/SrCO_3_ xerogels after annealing at 500 °C with various Sr/Al molar ratios: (**a**) 0.1, (**b**) 0.5, and (**c**) 1.0.

Figure 4 presents a comparison of the TEM micrographs of the Al_2_O_3_/SrCO_3_ samples with Sr to an Al ratio of 1.0 annealed at different temperatures. The black areas represent a strontium-rich phase (strontium carbonate), as confirmed by the XRD data presented in Figure 1a. The structures shown are typical for sol–gel systems [15].

**Figure 5:**
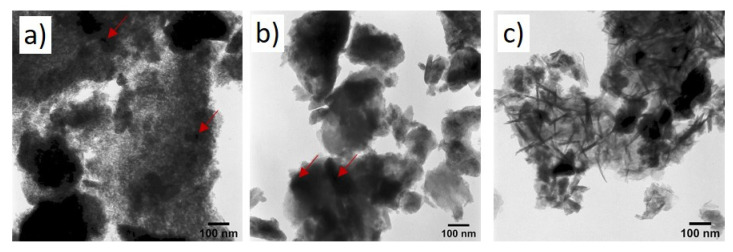
TEM images of Al_2_O_3_/SrCO_3_ sol–gel with Pt after annealing at 500 °C with molar ratios of: (**a**) Sr0.1 Pt, (**b**) Sr0.25 Pt, and (**c**) Sr0.5 Pt.

Figure 5 shows TEM images of Al_2_O_3_/SrCO_3_ sol–gel with Pt after annealing at 500 °C of samples with a molar ratio of Al/Sr 0.1, 0.25, and 0.5. The presented structures differ depending on the Al/Sr molar ratio. The Sr0.5Pt sample contains rod-shaped crystals, which are not observed in the other tested materials. The amorphous alumina structure is visible. The large dark fields show agglomerated strontium carbonate, and the smallest fields show platinum areas (marked with a red arrow).

### 2.3. Porous Structure—Low Temperature Nitrogen Adsorption–Desorption

**Figure 6:**
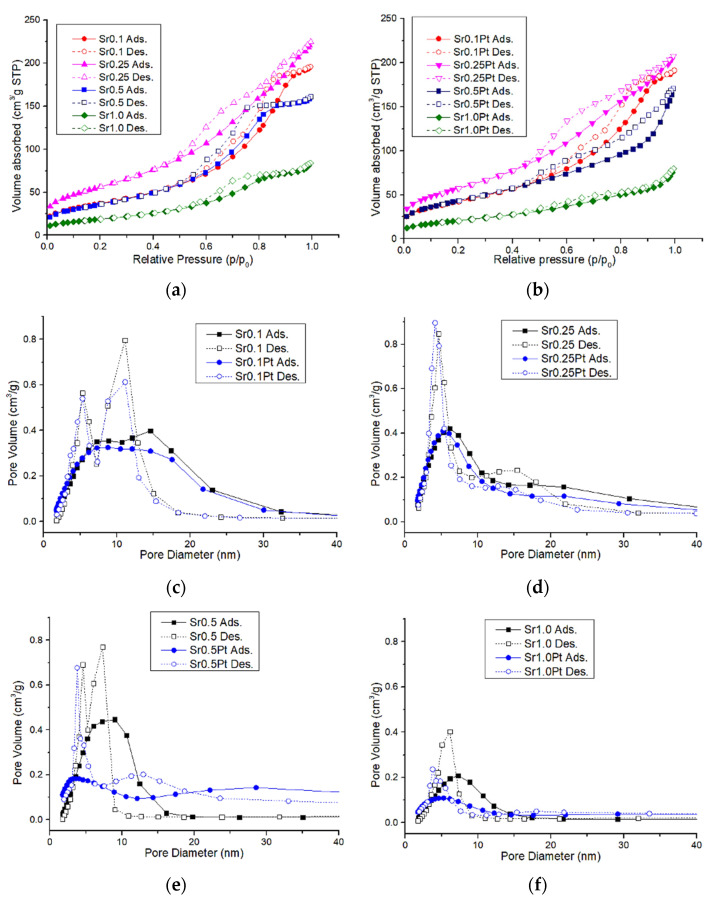
(**a**,**b**) Isotherm of Al_2_O_3_/SrCO_3_ and Pt/Al_2_O_3_-SrCO_3_, (**c**–**f**) pore volume distribution of Al_2_O_3_/SrCO_3_ and Pt/Al_2_O_3_-SrCO_3_.

Table 1 shows surface area, pore equivalent diameter, and volume. Figure 6 shows plots of isotherms, pore volume distribution, and pore area distribution. The adsorption isotherms for samples with different Sr/Al ratios are type IV with the hysteresis loop (IUPAC) present in the range of the relative pressure p/p_0_ 0.5–0.8, which is characteristic of mesoporous structures [24]. Additionally, all the analyzed samples are characterized by this isotherm. The shape of the hysteresis loops is similar for the samples with an Sr/Al ratio higher than 0.1. The adsorption isotherms for platinum samples differ from those with different Sr contents. Type III with the H_2_ hysteresis loop (except for the Sr1.0Pt system) is in the relative pressure range p/p_0_ 0.5–1.

The pore distribution curves depend on the desorptive branch of the BJH isotherm. In catalytic systems, the presence of scattered platinum on the surface leads to a slight increase in surface area (Table 1). In every system tested, the surface area of the Sr0.1 sample is twice that of a sample with an Sr/Al molar ratio of 1.0. Thus, the prepared Al_2_0_3_/SrCO_3_ xerogel samples exhibited specific surface areas above 100 m^2^/g (except for the Sr1.0 and Sr1.0 Pt). The surface area of the samples in which SrCO_3_ was used for the synthesis is greater compared to the samples with metal strontium. The average pore diameter is smaller for Al_2_O_3_/SrCO_3_ systems compared to the other samples. The average pore diameter and average pore volume are similar for the metallic strontium and platinum strontium systems. However, for systems with Sr/Al 0.5 and 1.0 molar ratios, a change in the geometry (a slight increase in the diameter) of the pores is visible, which may indicate the location of platinum crystals in the pores of the xerogel of a smaller size. For the systems with Pt, a slight increase in the specific surface area was also observed. This phenomenon may be related to the influence of platinum on the oxidative decomposition of carbon deposit residues that may occur in xerogel systems.

### 2.4. Chemisorption of Hydrogen on Pt-Al-Sr Catalysts

Table 2 shows hydrogen chemisorption results on Pt/Al_2_O_3_-SrCO_3_ systems with 1% metal content loading. Based on the results of hydrogen chemisorption, the platinum dispersion, the metallic surface area values, and the volume of the adsorbed hydrogen were determined. The results of hydrogen chemisorption for samples with a metallic strontium precursor showed no significant differences. The lowest metal dispersion occurred in the sample with an Sr/Al molar ratio equal to 1. Note that the Al_2_O_3_/SrCO_3_ system is alkaline. In such system (alkaline), dispersion is significantly lower, because the chemical nature of the surface of materials with large pores and low surface area is important. In our study, the alkaline nature of the system reduces platinum dispersion, but the presence of nanopores stabilizes the platinum nanocrystallites and, as a result, dispersion is beneficial.

### 2.5. Thermal Analysis

**Figure 7:**
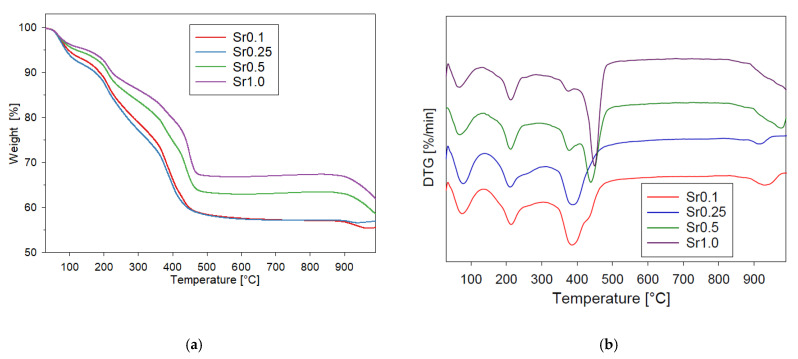
(**a**) TGA weight loss, and (**b**) DTG curves of Al_2_O_3_-SrCO_3_ xerogels obtained with a metallic strontium precursor.

For thermogravimetric testing, xerogel dried for one week at room temperature was used. Thermograms of the analyzed systems are shown in Figure 7a and the DTG curves in Figure 7b. Four visible areas of thermal changes in the xerogel were observed. The first step at temperatures up to 100 °C (373 K) is to remove the water adsorbed by the system. The second step of mass loss between 180 and 260 °C (453 to 533 K) was attributed to a correspondence to the removal of internally absorbed and trapped solvent residues and to the water of hydration in the gel. The third step of mass loss between 350 and 480 °C (623 to 753 K) can be attributed to the decomposition of anhydrous strontium acetate to SrCO_3_, as confirmed by the XRD results (Figure 1). In the sample Sr0.5 and Sr1.0, there is a fourth stage of weight loss above 900 °C (1173 K) with the decomposition of the strontium carbonate.

Furthermore, the decomposition processes can be described in the following steps [17,18,19,20,21,22,23,24,25]:

Sr(CH_3_COO)_2_ → SrCO_3 (s)_ + CH_3_COCH_3 (g)_ +CO_2 (g)_ (3)

SrCO_3 (s)_ → SrO _(s)_ + CO_2 (g)_ (4)

(5) $${\mathrm{Al}}_{2}O_{3}+\mathrm{SrO}\;\overset{>1150\;℃}{\to }\;{\mathrm{SrAl}}_{2}O_{4}$$

The DTG curves shown in Figure 7b indicate a multi-stage distribution. The decomposition of anhydrous strontium acetate to SrCO_3_ is shifted to the right as the strontium molar ratio increases.

## 3. Conclusions

In this study, a new method of obtaining binary Al_2_O_3_-SrCO_3_ xerogels systems is presented. The obtained xerogels are characterized by the presence of a stable carbonate phase for the full range of concentrations, which is confirmed by the XRD results. The effectiveness of the synthesis method using a reactive metallic precursor (metallic strontium) was confirmed. The obtained Al_2_O_3_/SrCO_3_ xerogels are characterized by a high dispersion of the carbonate phase and a large specific surface area for alkaline systems. Carbonate xerogels with an alkaline element are characterized by a similar dispersion of the metallic phase (42–50%) in all the tested systems, which is a very good result for alkaline systems. Changes in the nanoporosity system may confirm the theory of stabilization of platinum nanoclusters in the structure of nanopore carriers obtained by the sol–gel method. Xerogels obtained by the described method are also an attractive precursor for high-temperature ceramics with a strictly defined microstructure.

Carbonate xerogels with an element of basic nature are characterized by a similar dispersion of the metallic phase (42–50%) in all the tested systems, which is a very good result for alkaline systems.

Some strontium aluminates (such as SrAl₂O₄) are used as phosphors. Phosphors based on strontium aluminate are characterized by good luminescent properties such as long-lasting afterglow and high quantum efficiency in comparison to classic sulfide phosphors [26,27].

## 4. Materials and Methods

### 4.1. Materials

Strontium carbonate, aluminum isopropoxide, acetic acid, and toluene were purchased from Sigma-Aldrich (Saint Louis, MO, USA) and used as received.

### 4.2. Preparation

Al_2_O_3_-SrCO_3_ mixed systems with different molar ratios of Sr to Al: 0.1; 0.25; 0.5; and 1.0 were synthesized by aqueous sol–gel methods. Alumina gel was prepared according to our previous study [15]. Reactions were executed in a 1L glass reactor equipped with a mixer and a thermostat. Aluminum isopropoxide (500 g of fine powder) was added and hydrolyzed in 440mL of water at 75 °C. Then the obtained suspension was stirred for 2 h, and 175 g (167.5 cm^3^) of 98% acetic acid was peptized. The sol was heated under reflux for 24 h at 95 °C (368 K), followed by metallic strontium addition in small portions as the second component. The next step was refluxing the resulting mixture for 72 h with vigorous stirring. The obtained product was a homogeneous liquid gel. To obtain a monolithic xerogel, part of the obtained gel was spilled into Petri dishes and dried for 5 days at room temperature. Next, the dry gel was annealed in a tube furnace at 500 °C (773 K) for 6 h under air flow. Part of the sample was air dried for 3 h, then annealed in 1000 °C (1273 K). Next, a portion of the sample was taken and air dried for 3 h and annealed in 1150 °C (1423 K). Finally, the sample batch was air dried for 3 h and annealed in 1300 °C (1573 K). The sample obtained after the annealing was crushed and sieved. Two grain fractions were collected: 0.1–0.2 mm and <0.1 mm. The particle size fraction with a diameter of 0.1 to 0.2 mm was used to determine the porous structure.

To prepare the Pt catalysts, 1.98 g of the support annealed at 500 °C (773 K) was weighed and placed in a 100 mL round-bottom flask. The samples were wetted with 2 mL of distilled water, then 1 mL of aqueous H_2_PtCl_6_ solution (platinum content: 20 mg Pt/mL) was added. The round-bottom flask was placed on a vacuum evaporator, and the solvent was evaporated. The dried system was heat treated at a temperature of 500 degrees for 2 h in air atmosphere.

**Figure 8:**
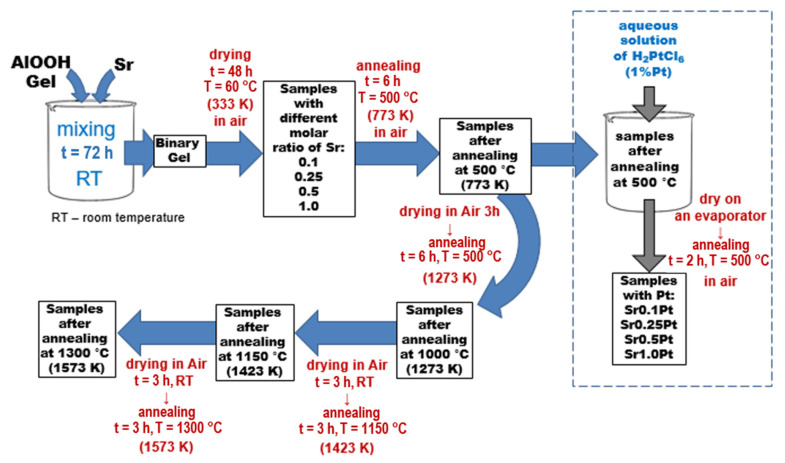
Schematic procedure of Al_2_O_3_/SrCO_3_ xerogels synthesis.

Figure 8 shows the scheme of the synthesis of Al_2_O_3_/SrCO_3_ xerogels.

### 4.3. Characterization

The samples obtained in this work were characterized by the following techniques.

#### 4.3.1. X-ray Diffraction Analysis

The X-ray diffraction (XRD) analysis was performed using the Philips PW1050 diffractometer (Almelo, the Netherlands) working in the θ–2θ geometry with Cu-K_α_ (λ = 0.15406 nm) radiation of 35 kV and 20 mA. For all the samples, an angular range (2θ) of 10° to 100° with a step width of 0.01° and a step time of 3 s was used [28].

#### 4.3.2. SEM, TEM, and EDS (Energy Dispersive X-ray Spectroscopy) Analysis

The surface morphology of the oxide xerogels was depicted by a Scanning Electron Microscope (SEM, Hitachi 3000N, Tokyo, Japan), which was operated in high-vacuum conditions at 15 kV acceleration voltage and low-vacuum conditions at 20 kV acceleration voltage. Chemical composition was performed using the Energy Dispersive Spectroscopy (EDS). TEM observations were performed using a JEOL 200 CX (Tokyo, Japan) transmission electron microscope worked at 80 kV.

#### 4.3.3. Porous Structure

In order to determine the porosity of the structure, measurements of low-temperature nitrogen adsorption were carried out using the Autosorb iQ Station 2 (Quantachrome Instruments, Boynton Beach, Florida, United States) in the standard analysis mode; 200–300 mg of material with a particle size fraction from 0.1 to 0.2 mm were tested. Prior to testing, the samples were degassed for 10 h at 350 °C (623 K) and 0.4 Pa to constant weight. The adsorption and desorption isotherm branches were assumed in the p/p0 0–1 range. Quantachrome ASiQwin software (version 2.0) was used. The Boer t-method and the BJH method were used to determine the distribution of the pore surface and pore volume. The volume and diameter of the pores were determined by the BJH method from the adsorption branch of the isotherm. The BET multipoint linear regression method was used to calculate the surface area using the p/p0 0.1–0.3 window and the available seven degrees of freedom (nine data points) [25].

#### 4.3.4. Thermal Analysis

On the NETZSCH TG 209 F1 Libra thermogravimeter (Selb, Germany), the thermal conversion of unprocessed (air-dried) samples was carried out. Five mg of the sample was placed in an alumina vessel (volume 85 µL) and heated from 20 °C·min^−1^ to 1000 °C (1273 K). For analysis, the fraction with a grain size <0.1 mm was used. TG traces were recorded with air flow (20 cm^3^·min^−1^) with a resolution of 0.1 µg. Drying under vacuum or at elevated temperature was not applied [28].

#### 4.3.5. Chemisorption of Hydrogen on Pt-Al-Sr Catalysts

Hydrogen chemisorption was carried out by means of an ASAP 2010C sorptometer (Micromeritics, Norcross, GA, USA). Samples had previously been reduced with H_2_ at 400◦C (673 K) during 2 h. Then the samples were evacuated in a sorptometer at room temperature for 0.25 h, then at 350 °C (623 K) for 1 h. After 1 h, the samples were reduced with a hydrogen flow (2.4 L/h) at 350◦C (623 K) and degassed for 2 h at 350◦C (623 K). Hydrogen chemisorption studies were performed at 35 °C (308 K). The platinum dispersion was determined from the total amount of chemically adsorbed hydrogen. The following equation was used to calculate the metal surface area S [29]:

(6) $$S=\frac{v_{m}\cdot N_{A}\cdot n\cdot a_{m}\cdot 100}{22414\cdot m\cdot wt}\left[m^{2}\cdot g_{Pt}^{-1}\right]$$

where *v_m_*—volume of adsorbed hydrogen (cm^3^), *N_A_*—Avogadro’s number (6.022 × 1023 mol^−1^), *n* = 1 is the chemisorption stoichiometry, *wt* (%)—the metal loading, *a_m_*—the surface area (m^2^), and *m*—the sample mass (g). The following formula was used to calculate the dispersion of the active phase:

(7) $$D=\frac{S\cdot M}{a_{m}\cdot N_{A}}$$

where *S* is the metal surface area, *M* is the platinum atomic weight, *N_A_* is Avogadro’s number, and *a_m_* is the surface occupied by one platinum atom.

## Figures and Tables

**Table 1:** Textural properties of Sr systems.

System Composition	Surface Area S_BET_ [m^2^/g]	Average Pore Diameter D_BJH_ [nm]	Average Pore Volume D_BJH_ [cm^3^/g]
Sr0.1	135	7	0.30
Sr0.25	204	6	0.34
Sr0.5	132	6	0.25
Sr1.0	69	6	0.13
Sr0.1 Pt	154	7	0.29
Sr0.25 Pt	209	6	0.31
Sr0.5 Pt	159	7	0.25
Sr1.0 Pt	75	6	0.12

**Table 2:** Platinum dispersion, surface area, and volume of adsorbed hydrogen of the Pt/Al_2_O_3_-SrCO_3_ systems.

System Composition	Metal (Pt) Dispersion [%]	Metallic Surface [m^2^/g_metal_]	Volume of Adsorbed Hydrogen [cm^3^/g]
Sr0.1 Pt	47	116.94	0.27 ± 0.006
Sr0.25 Pt	49	122.55	0.29 ± 0.004
Sr0.5 Pt	48	119.15	0.28 ± 0.001
Sr1.0 Pt	42	102.94	0.24 ± 0.003

## Data Availability

Not applicable.
